# Formulation and evaluation of floating tablets of liquorice extract

**DOI:** 10.4103/0974-8490.72329

**Published:** 2010

**Authors:** H. N. Aswatha Ram, Prachiti Lachake, Ujjwal Kaushik, C. S. Shreedhara

**Affiliations:** *Department of Pharmacognosy, Manipal College of Pharmaceutical Sciences, Manipal University, Manipal - 576 104, Karnataka, India*

**Keywords:** Buoyancy time, floating tablets, korsemeyer, liquorice extract

## Abstract

**Background:**

Floating tablets prolong the gastric residence time of drugs, improve bioavailability, and facilitate local drug delivery to the stomach. With this objective, floating tablets containing aqueous extract of liquorice as drug was prepared for the treatment of *Helicobacter pylori* and gastric ulcers.

**Methods:**

The aqueous extract of liquorice was standardized by HPTLC. Tablets containing HPMC K100M (hydrophilic polymer), liquorice extract, sodium bicarbonate (gas generating agent), talc, and magnesium stearate were prepared using direct compression method. The formulations were evaluated for physical parameters like diameter, thickness, hardness, friability, uniformity of weight, drug content, buoyancy time, dissolution, and drug release mechanism. The formulations were optimized on the basis of buoyancy time and *in vitro* drug release.

**Results:**

The diameter of all formulations was in the range 11.166–11.933 mm; thickness was in the range 4.02–4.086 mm. The hardness ranged from 3.1 to 3.5 kg/cm^2^. All formulations passed the USP requirements for friability and uniformity of weight. The buoyancy time of all tablet formulations was less than 5 min and tablet remained in floating condition throughout the study. All the tablet formulations followed zero-order kinetics and Korsemeyer-Peppas model in drug release.

**Conclusion:**

The optimized formulation was found to be F6 which released 98.3% of drug in 8 h *in vitro*, while the buoyancy time was 3.5 min. Formulations containing psyllium husk, sodium bicarbonate and HPMC K100M in combination can be a promising for gastroretentive drug delivery systems.

## INTRODUCTION

Oral controlled release dosage forms have been developed over the past three decades due to their considerable therapeutic advantages such as ease of administration, patient compliance, and flexibility in formulation. However, this approach has several physiological difficulties such as inability to restrain and locate the controlled drug delivery system within the desired region of the gastrointestinal tract (GIT) due to variable gastric emptying and motility. Gastroretentive dosage form can remain in the gastric region for several hours and hence significantly prolong the gastric residence time of drugs. Prolonged gastric retention improves bioavailability, reduces drug waste, and improves solubility of drugs that are less soluble in a high pH environment. The types of gastroretentive dosage forms are floating drug systems – effervescent and noneffervescent systems.[1]

Liquorice consists of dried peeled or unpeeled roots and stolons of *Glycyrrhiza glabra* Linn belonging to the family Fabaceae.[2] It has been reported that liquorice is effective in gastric ulcer treatment[3] and glycyrrhetinic acid, the aglycone of glycyrrhizin, has an anti-inflammatory and antiulcer effect.[4] Liquorice can raise the concentration of prostaglandins in the digestive system that promote mucus secretion from the stomach; it was also reported that liquorice prolongs the life span of surface cells in the stomach and has an antipepsin effect.[5] It has also been reported that *Helicobacter pylori* shows susceptibility to liquorice extract.[6]

## MATERIALS AND METHODS

*Materials*. The roots and rhizomes of the plant *Glycyrrhiza glabra* were purchased from local market in Udupi, India. The botanical identity was confirmed by Professor V. Aravinda Hebbar, Department of Botany, Mahatma Gandhi Memorial College, Udupi, India. HPMC K100M was obtained from Rolex Laboratory, Hyderabad. Psyllium husk was purchased from a local market in Udupi, India. Sodium bicarbonate was obtained from Sisco Research Laboratories Pvt. Ltd., Mumbai. Talc was obtained from Swastik Pharmaceuticals, Mumbai. Magnesium stearate was obtained from Modern Chemical Corporation. 18-β-Glycyrrhetinic acid was procured from Sigma Aldrich Inc., USA. Chloroform was obtained from Nice Chemicals Pvt. Ltd., Cochin. Formic acid was obtained from Chemicals Pvt. Ltd., Cochin. Acetone, diethyl ether, and methanol were obtained from Merck Specialties Pvt. Ltd., Mumbai. All chemicals used were of analytical and pharmaceutical grade.

*Preparation and standardization of aqueous extract from liquorice root*. The powdered liquorice root was extracted with distilled water containing ammonia. The extraction temperature was maintained at 90°C with constant shaking. The extract was filtered and concentrated to get a thick paste. The amount of glycyrrhetinic acid in the extract was determined by HPTLC.[7]

*Sample preparation*. Two hundred and fifty milligrams of the extract was refluxed with 50 mL 1N HCl for 4 h. It was cooled to room temperature and it was extracted with (20 × 5) mL chloroform. The combined chloroform extract was washed with water and filtered. It was evaporated at temperature of 30°C and the residue was dissolved in chloroform: methanol (1:1) and the volume was made up to 25 mL.

*Standard preparation*. Ten milligram of 18-β-glycyrrhetinic acid was dissolved in 25 mL of chloroform: methanol (1:1).

**Table:** 

*Mobile phase.*	First run	chloroform-acetone (9:1)
	Second run	chloroform-diethyl etherformic acid (80:15:1)

*Method*. 2, 3 and 4 µL of standard and sample were applied in bands on precoated silica gel HPTLC plates by Linomat V applicator. Band length was 6 mm and distance between the bands was kept at 4 mm. The plate was dried and scanned at 254 nm in absorbance mode. The amount of glycyrrhetinic acid was found by comparison of peak area of standard and extract. The results are given in Table 1.

**Table 1:** HPTLC of liquorice extract

Track	Peak	R_f_	Peak area	Volume µL
1	1	0.23	5139.3	2
2	1	0.21	4801.7	2
3	2	0.2	6454	3
4	2	0.2	7763	4
5	2	0.18	743.8	2
6	2	0.18	1111.6	3
7	2	0.18	1309.9	4	

*Formulation of tablets*. In the present study, all the tablets were formulated by direct compression technique using polymer like HPMC K100M and other ingredients like psyllium husk, magnesium stearate, talc, and sodium bicarbonate. All ingredients were passed through sieve no # 80 and weighed accurately on electronic balance. The extract, HPMC K100M, sodium bicarbonate, and psyllium husk were mixed properly in a mortar and pestle to get a uniform tablet blend. Finally talc and magnesium stearate were mixed with the blend. The tablet blend was then weighed individually according to the formula and compressed into tablets using single punch tableting machine (Cadmach, Ahmedabad). The different formulations were labeled F1–F7 and their formulae are given in Table 2.

**Table 2:** Composition of floating tablet formulations

Ingredients mg	F1	F2	F3	F4	F5	F6	F7
Liquorice extract	250	250	250	250	250	250	250
Psyllium husk	75	100	125	100	100	100	100
HPMC K100M	50	50	50	40	60	50	50
Sodium bicarbonate	100	100	100	100	100	90	110
Talc	20	20	20	20	20	20	20
Magnesium stearate	5	5	5	5	5	5	5

*Evaluation of floating tablets*. The prepared floating tablets were evaluated for diameter and thickness using Vernier calipers. The hardness of the tablets was evaluated using a Monsanto hardness tester. The friability was determined in a Roche friabilitor. Twenty tablets from each formulation were weighed and their average weight was determined [Table 3].

**Table 3:** Evaluation of formulated tablets

Formulation	Diameter (mm)	Thickness (mm)	Hardness (kg/cm^2^)	Friability (%)	Uniformity of weight (mg)	Drug content (%)	Buoyancy time (minutes)
F1	11.166±0.115	4.02±0.01	3.133±0.251	0.85±0.032	500.4±0.683	97.273±0.499	5±0.288
F2	11.533±0.208	4.066±0.011	3.1±0.2	0.868±0.022	525.3±0.575	98.026±0.902	4.5±0.5
F3	11.933±0.152	4.086±0.005	3.4±0.264	0.726±0.045	550.665±0.745	98.277±0.662	4±0.763
F4	11.3±0.1	4.036±0.025	3.366±0.23	0.716±0.093	515.285±0.736	99.61±0.631	4±0.866
F5	11.733±0.115	4.073±0.015	3.333±0.208	0.804±0.076	535.16±0.752	97.442±0.521	5±0.577
F6	11.4±0.2	4.046±0.02	3.466±0.057	0.811±0.091	515.22±0.689	96.107±0.382	3.5±0.5
F7	11.7±0.264	4.07±0.02	3.5±0.1	0.675±0.043	535.235±0.605	99.527±0.661	5±0.763

*Buoyancy time*. The time taken for dosage form to emerge on surface of medium called floating lag time (FLT) or buoyancy lag time (BLT). Floating behavior studies were performed in a USP type II (paddle) apparatus at speed 100 rpm in 900 mL 0.1N HCl at 37 ± 0.2°C to mimic *in vivo* conditions. FLT was determined on the basis of visual inspection.[8]

*In vitro dissolution studies*. The in vitro dissolution studies were carried out using USP type I (basket) apparatus. The dissolution medium was 900 mL 0.1N HCl. The dissolution medium was kept in thermostatically controlled water bath, maintained at 37±0.5°C. The tablet was placed into the basket. The speed of rotation was kept at 100 rpm. At different time intervals, 5 mL of sample was withdrawn and dissolution medium was kept constant throughout by replacing with equal volume 5 mL of dissolution medium. The aliquots were extracted with 30 mL of chloroform and the chloroform fraction was analyzed spectrophotometrically (Shimadzu UV 1650PC) at 251 nm against blank chloroform for drug release. The study was performed in triplicate. A plot of cumulative % drug release versus time in hours was plotted.[8]

*Analysis of release kinetics*. The mechanism of release was determined by fitting the release data to the various kinetic equations such as first-order,[9] zero-order, Higuchi,[10] and Korsmeyer-Peppas[11] and the R2 values of the release profile corresponding to each model were found. The results are shown in Table 4.

**Table 4:** Analysis of release mechanism

Formulation	Zero order R^2^	First order R^2^	First order k (h^-1^)	Higuchi R^2^	Korsemeyer R^2^	Korsemeyer n
F1	0.9668	0.9049	0.2025	0.9794	0.9973	0.6893
F2	0.9751	0.9219	0.1596	0.9738	0.9904	0.6788
F3	0.9507	0.7858	0.3085	0.9908	0.9973	0.6241
F4	0.945	0.9379	0.2234	0.9865	0.9949	0.635
F5	0.9698	0.9361	0.2062	0.9714	0.9954	0.7177
F6	0.9503	0.9161	0.2445	0.9799	0.9817	0.7295
F7	0.9912	0.9241	0.1751	0.9523	0.9982	0.8408

## RESULTS

HPTLC of the extract. The content of glycyrrhetinic acid present in the extract by HPTLC was found to be 18.3745% w/w.

*Evaluation of formulated tablets*. The diameter of all formulations was in the range 11.166-11.933 mm; thickness was in the range 4.02-4.086 mm. The hardness ranged from 3.1 to 3.5 kg/cm^2^. All formulations passed the USP requirements for friability and uniformity of weight [Table 3].

*Buoyancy time*. The buoyancy time of formulations are shown in Table 3. FLT of all formulations was found to be less than 5 min. The carbon dioxide generated from sodium bicarbonate upon contact with the acidic medium will remain entrapped in the gellified layer of the swollen polymer (hydrocolloids). This produces an upward motion of the dosage form and maintains its buoyancy.[12,13] The FLT may be explained as a result of the time required for dissolution medium to penetrate the tablet matrix and develop the swollen layer for entrapment of CO_2_ generated *in situ*. The tablet mass decreased progressively due to liberation of CO_2_ and release of drug from the matrix. On the other hand, as solvent front penetrated the glassy polymer layer, the swelling of HPMC K100 M caused an increase in volume of the tablet. The combined effect is a net reduction in density of the tablets, which prolongs the duration of floatation beyond 8 h.

In the present study for the development of floating tablets two approaches were used - to optimize floating behavior of the dosage form for 8 h and to optimize the release characteristics of the drug which could afford to release 99% of the drug for about 8 h.

During formulation development, the ingredients used were selected based on the approach of achieving drug release for 8 h. Floating drug delivery is based on the swelling property and density of the polymers as well as the gas generating agent.[14] The work was started using psyllium husk which swells upto 14 times of its original volume and the density of psyllium husk is lower than the gastric fluids.[15] Sodium bicarbonate was used as a gas-generating agent, which reacts with the gastric fluids and produces carbon dioxide. This gas is entrapped into swollen matrix and provides buoyancy to the formulation.[14] HPMC K100M having high viscosity and ability to swell was used.

**Figure 1:**
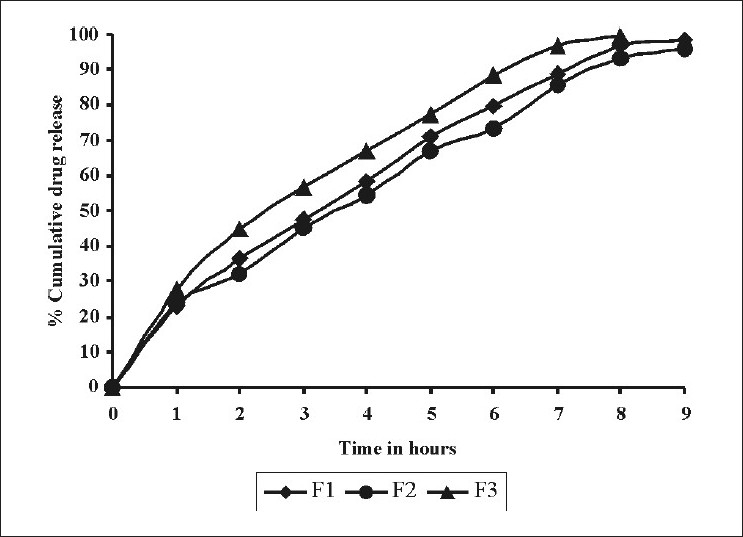
Comparative drug release profile of tablet formulations F1, F2 and F3

In vitro drug release. In this work, we have carried out in vitro drug studies in 0.1N HCl as the dissolution medium to study the drug release of the tablet formulations. Effect of different concentrations of psyllium husk on in vitro release was as shown in Figure 1. As the concentration of psyllium husk increased from 75 (F1) to 125 mg (F3) per tablet, the percent cumulative drug release decreased from 98.29 ± 0.86% (F1) to 96.1 ± 0.634% (F2). The percent cumulative drug release for (F3) was 99.8 ± 0.965% after 8 h. The slow release of the drug could have attributed to the gelling properties of psyllium husk.

**Figure 2:**
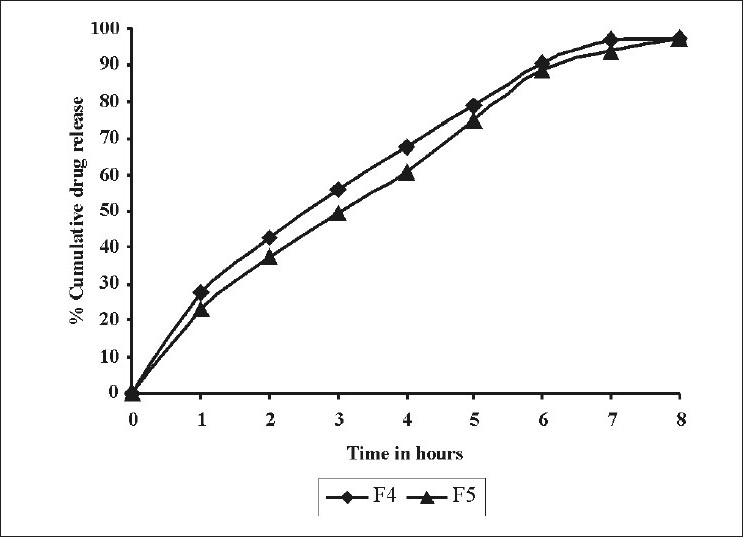
Comparative drug release profile of tablet formulations F4 and F5

Effect of different concentrations of HPMC K100M on in vitro release was as shown in Figure 2. As the concentration of HPMC K100M was increased from 40 (F4) to 60 mg (F5), drug release decreased from 97.5± 0.696% to 97.3 ± 0.408%. This might be due to the increased polymer concentration which could have increased the diffusion path length for the drug, which could have retarded the drug release.

**Figure 3:**
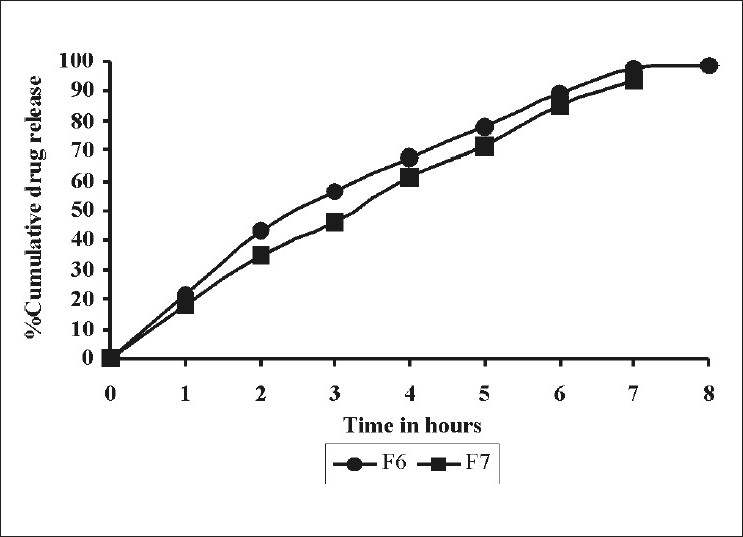
Comparative drug release profile of tablet formulations F6 and F7

The effect of sodium bicarbonate on in vitro drug was shown in Figure 3. In such systems, sodium bicarbonate acts as a gas-generating agent. It generates gas when it comes into contact with an acidic environment of the stomach. This gas entraps into the matrix of water-soluble polymers and the formulation floats in an acidic environment of the stomach. As the concentration was increased from 90 (F6) to 110 mg (F7) per tablet, the drug release was decreased from 98.3 ± 0.935% to 93.6 ± 0.706%. Sodium bicarbonate being alkaline in nature creates an alkaline microenvironment around the tablet, which decreased the drug release from the tablet.

Optimization of tablet formulation. Based upon the buoyancy time and % cumulative drug release formulations were optimized. The buoyancy time of all formulations was in the range 3.5-5 min. The % cumulative drug release was in the range 93.34-99.8%. The optimized formulation was found to be F6. The buoyancy time was 3.5 min and % cumulative drug release was 98.3%.

*Analysis of release kinetics*. To study the release rate kinetics and the release mechanism of the drug from the tablet formulations, the in vitro drug release data were treated with the mathematical equation such as first order kinetics equation, zero-order kinetics equation, Higuchi’s equation, and Korsemeyer’s equation. The data obtained are represented in Table 4. When data were treated with Higuchi’s equation to learn about the mechanism of drug release, it was observed that the values did not give a good fit for the Higuchi equation. None of the formulations followed first-order kinetics, which was confirmed by the poor correlation coefficient values. All formulations best fitted both zero-order (R^2^ =0.945–0.9912) and Korsemeyer and Peppas equation (R2 =0.9817-0.9982). When n takes value 0.5, it indicates Fickian diffusion controlled drug release and for the value 1.0 indicates case II transport (swelling-controlled drug release). Values of n between 0.5 and 1.0 can be regarded as an indicator for the non-Fickian (anomalous transport) diffusion. For all formulations, the value of n was in the range 0.6242-0.8408 indicating anomalous transport wherein the drug release mechanism is controlled by both diffusion and polymer relaxation.

## CONCLUSION

Floating tablets of liquorice extract using psyllium husk, HPMC K100M, talc, sodium bicarbonate, and magnesium stearate were prepared. Formulated tablets were within acceptable limits for various physicochemical evaluations for tablets like tablet dimensions, hardness, uniformity of weight, friability, buoyancy time, and in vitro drug release. *In vitro* dissolution studies for the floating tablets were carried out in 0.1N HCl at 37 °C. About 93–99% of the drug was released in 8 h. Formulation F6 showed good floating behavior along with better--controlled drug release in comparison to other prepared formulations. Formulated floating tablets best fitted to Korsmeyer-Peppas model and zero-order kinetics. All formulations for the value of n was in the range 0.624-0.84 indicating anomalous transport wherein the drug release mechanism is controlled by both diffusion and polymer relaxation. We can conclude that psyllium husk, sodium bicarbonate and HPMC K100M in combination can be promising polymers for gastroretentive drug delivery systems. Floating tablets of aqueous extract of liquorice can be formulated as an approach to increase gastric residence time, thereby improving its bioavailability. The results indicate a promising potential of aqueous extract of liquorice floating tablets as an alternative to the conventional dosage form.
